# Engagement with body image health promotion videos in adult men and women: differences between narrative, informational, and persuasive appeal approaches

**DOI:** 10.1186/s40359-023-01120-7

**Published:** 2023-03-29

**Authors:** Jo R Doley, Siân A McLean

**Affiliations:** 1 Institute for Health and Sport, Victoria University, PO Box 14428, Melbourne, VIC 8001 Australia; 2 School of Psychology and Public Health, La Trobe University, Melbourne, VIC 3086 Australia

**Keywords:** Persuasive communication, Engagement, Social marketing, Body dissatisfaction

## Abstract

**Background:**

Body dissatisfaction is a public health issue, however, low awareness of its seriousness, and stigma, may inhibit treatment seeking. The current study evaluated engagement with videos promoting awareness of body dissatisfaction using a persuasive communication approach.

**Method:**

Men (*n* = 283) and women (*n* = 290) were randomly allocated to view one of five videos; (1) Narrative, (2) Narrative plus persuasive appeal, (3) Informational, (4) Informational plus persuasive appeal and (5) Persuasive appeal only. Engagement (relevance, interest, and compassion) was examined post-viewing.

**Results:**

Among both men and women, superior engagement ratings (in compassion for women, and relevance and compassion for men) were demonstrated for the persuasive appeal and informational videos relative to narrative approaches.

**Conclusion:**

Videos using clear and factual approaches may promote engagement in body image health promotion videos. Further work should be done to examine interest in such videos specific to men.

**Supplementary Information:**

The online version contains supplementary material available at 10.1186/s40359-023-01120-7.

## Introduction

Body dissatisfaction, negative evaluation of one’s appearance, shape, or weight, poses a serious public health issue [1]. It is associated with depressive mood [2] and eating disorders [3], and leads to poor health outcomes in multiple domains [4–6]. Little research has examined the public’s awareness of body dissatisfaction as a serious mental health issue, but the research that exists finds that mental health literacy around the problem is very low. For example, one study demonstrated that 65.2% of women and 34.8% of men indicated that there was nothing wrong with the behaviour (e.g., social comparison, avoiding social events) exhibited by a character in a vignette with body dissatisfaction and only 18.4% of participants in the same study correctly identified that the character’s mental health problem was body dissatisfaction [7]. Additionally, a qualitative study identified that participants at an exercise facility had poor understanding of the construct of body image, and sometimes conflated a thinner appearance or weight loss with positive body image [8]. Similarly, poor understanding of the consequences of body dissatisfaction for men were identified among male participants [9]. As body dissatisfaction mental health literacy is generally low in the population, it is important that evidence-based, wide-reaching campaigns about this problem are available to facilitate appropriate treatment-seeking [10]. This study aimed to evaluate the effects of viewing short video messages for social marketing campaigns – specifically, examining engagement with those messages – about body dissatisfaction through a lens of persuasive communication.

Social marketing campaigns are becoming increasingly common within mental health [11–13], including body dissatisfaction [14, 15]. A common technique in such campaigns is persuasive communication [16–18]. Persuasive communication is communication that is designed to change, produce, or influence attitudes and behaviours [19, 20]. Previous research has demonstrated that persuasive communication techniques have produced positive changes in body image [21], and attitudes towards eating disorders [22] and disordered eating behaviours [23]. While several studies have evaluated the effectiveness on individual attitudes or knowledge, research evaluating broader applications of such campaigns [10], such as whether viewers find the campaigns engaging, or whether the campaigns motivate them to share or learn more about the issue, is lacking.

## Theoretical background

### Social marketing engagement

One essential component of social marketing campaigns is that they are *engaging*. Social marketing engagement is a relatively recent area of research, and as such the literature is not yet extensive. Definitions of social media or social marketing engagement have typically come from research around brands. It is important to note that, within the context of this research and its applications, the term “brand” is applied broadly, and inclusive of non-government organisations and academic institutions, rather than solely focusing on capitalist consumer brands. While there is no one definition of engagement, there are commonalities in theoretical models of the construct [24–27]. Models of engagement define engagement as having cognitive, affective, and behavioural [28] components; with some including social components [27]. Affective components refer to the consumer’s emotional response to the content (e.g., enthusiasm, anger), behavioural components refer to the consumer’s behavioural response to the content (e.g., sharing, liking) and cognitive components refer to the thoughts the consumer has in response to the content (e.g., processing the content in regards to one’s own life experiences; [24, 25]. The less commonly examined construct, the social component of engagement, refers to co-creation of content (e.g., photographs, memes) and interaction with a brand [26]. As such, it is agreed that engagement is a multidimensional construct [24], and high engagement contributes beneficial outcomes for social marketing in that it promotes dissemination and discussion of information, rather than simply viewing information [29, 30]. Generally, engagement may be active (e.g., sharing content, liking content, co-creating content) or more passive (e.g., consuming content; [31, 32]. As the social component of co-creation and interaction would not necessarily be possible with a one-off video in an experimental study, engagement is agreed to include affective, behavioural, and cognitive components [24, 25], the current study focused on these three domains.

### Persuasive communication

The type of communication that may result in greater engagement in a message has not been investigated within the context of body image. Previous research has identified two broad types of persuasive communication; narrative and informational [33, 34]. Informational communication conveys statistics, facts, or scientific information [33, 35]; for instance, an informational campaign about body dissatisfaction may include information about the characteristics of body dissatisfaction and the demographics most affected. An informational approach relies on the assumption that people generally process information using logic and reasoning. Narrative communication uses a storytelling approach with defined characters and may be fictional or non-fictional – as highlighted by Hinyard and Kreuter [33] there is great variation in the definition of narrative communication in the literature. As such, they proposed that “A narrative is any cohesive and coherent story with an identifiable beginning, middle, and end that provides information about scene, characters, and conflict; raises unanswered questions or unresolved conflict; and provides resolution.” (p.778). For instance, a narrative story about body dissatisfaction may portray a person experiencing body dissatisfaction who discusses their negative thoughts and behaviours (e.g., being distressed about their weight or shape), include questioning the usefulness of these thoughts and behaviours, and resolve with the person being more self-compassionate about their body.

The effectiveness of both informational and narrative approaches has been found elsewhere throughout the literature with small effects on behavioural and attitudinal outcomes in meta-analyses [35, 36]. It has been identified that narrative communication may be less resistant to counterarguing – whereby viewers raise arguments or objections to the premise of a persuasive message – than informational communication [37], as the focus is on experiences and feelings of characters. Neither persuasive style has been evaluated for engagement per se, however one study identified that non-narrative information was more likely to result in elaboration of the arguments presented [38].

Persuasive health messages may also contain persuasive appeals, which are intended to clarify the content and meaning of narrative communication [11, 18, 39]. For instance, an episode of a television program about drink driving contained a message explicitly explaining the danger of binge drinking and drink driving to serve as a clarification of a narrative that depicted an incident of drink driving [18]. Inclusion of such appeals is intended to further reduce counterarguing or misinterpretation of the message and have shown to be effective when combined with narrative information [17, 40, 41]. As informational approaches may be less open to individual interpretation compared with narrative approaches, it is unsurprising that adding persuasive appeals to informational communication has not yet been investigated. However, the inclusion of a direct persuasive appeal may increase engagement with informational messages, which could otherwise be perceived as dry and bland. Additionally, to the authors’ knowledge, the effect of a persuasive appeal alone has not been investigated relative to informational or narrative approaches. A persuasive appeal alone may be an effective approach for a social media or general media audience, to attempt to capture attention in a social media environment with a great deal of competing content.

### Impact of persuasive approaches on domains of engagement

While no literature exists examining the impacts of persuasive communication approaches on engagement, previous work suggests that narrative and informational approaches persuade using different mechanisms [17, 33, 42]. As such, they may impact different domains of engagement. The narrative approach, with its focus on experiences and feelings of characters [33, 35, 41], theoretically may influence the affective domain more strongly than the cognitive or behavioural; whereas the informational approach, with its focus on statistics and reasoning [33, 35], may promote more central or systematic processing of messages and thus may be more likely to impact the cognitive domain over others. Hinyard and Kreuter [33] elaborate on the manner through which narrative approaches are thought to persuade people, describing that typically they are approached through hedonic processing (i.e. entertainment). Whether the addition of a persuasive appeal may influence a particular outcome is unknown. Some empirical work on attitude change suggests different persuasive messages may have differing outcomes on attitude change [43, 44], also suggesting different mechanisms of action. For instance, previous research on the effect of attitude bases (i.e., whether attitudes are thought to be driven more by cognitive or affective components) suggest that cognitive (which is primarily informational) and affective (an important component of narratives) messages may impact attitude change differently [45]. However, there are mixed findings on whether a match or a mismatch between attitude basis and message type is best for persuasive impact [43–45]. Additionally, this research has focused on attitude change rather than engagement.

Identifying whether particular domains of engagement may be particularly useful for targeted campaigns; for instance, campaigns aiming to promote socio-political change, may benefit from changing behavioural engagement, while campaigns aiming to reduce stigma may wish to impact cognitive and affective domains. As such, this study also sought to understand whether the type of persuasive communication may impact different engagement domains.

### Current study

Little research in body dissatisfaction and eating disorders has identified the specific approach taken in persuasive communications as either informational or narrative [22]. In our earlier analysis of a separate element of the current study, it was found that informational communication had stronger effects for improving perceptions of the seriousness of body dissatisfaction than narrative messages, whereas narrative and informational approaches, with or without a persuasive appeal, were equivalent in producing positive effects for reducing body dissatisfaction in viewers [46]. The level of engagement in such messages has not yet been investigated. Outside the body image sphere, both informational [47] and emotionally engaging (e.g., which may come from a narrative approach) content from brands result in users sharing posts [48, 49], but these approaches have not been compared directly. Additionally, we sought to compare these approaches separately for men and women, as attitudes around body dissatisfaction tend to differ between genders. For instance, men tend to attribute greater blame for illness towards those with eating disorders than do women and have poorer recognition of symptoms of eating disorders [50] and body dissatisfaction [7] than women.

As social media (and increasingly, traditional media with a social media presence) relies heavily on sharing of information (e.g., sharing a health message campaign to one’s own social media account, or through email) and engagement (e.g., comments, likes, emotional investment in the topic), the current study sought to understand the effect of different types of persuasive communication on engagement in a body image social marketing video. Thus, the current study aimed to examine the extent to which such a campaign can generate cognitive, affective, and behavioural engagement and to compare levels of engagement across informational, narrative, and persuasive appeal communication approaches. As engagement has not previously been investigated within this context, no specific hypotheses were made. However, the following research questions were explored:

RQ1: Of the persuasive approaches (informational, narrative, persuasive appeal, informational and persuasive, narrative and persuasive), which results in superior engagement in the topic of body dissatisfaction mental health literacy?

RQ2: Do particular persuasive communication approaches impact the cognitive, affective, and behavioural domains of engagement differently?

## Method

### Participants

Participants were recruited through Prolific, an online participant platform. Men and women aged 18–45 from Australia, Canada, or the United Kingdom were eligible to take part in the study. Participation was limited to persons from these countries as they are culturally similar to Australia, in which the stimulus materials were filmed. Initially, 633 participants responded to the study. After removing participants who chose to withdraw their data following debriefing where they learned the true aims of the study (*n* = 24), failed the attention check (*n* = 3), were ineligible (had seen the stimulus video previously, *n* = 8), or did not provide responses after watching the video (*n* = 25) that would exclude them from analyses, a final sample of *N* = 573 (*n* = 116 narrative only, *n* = 109 narrative + persuasive appeal, *n* = 118 persuasive appeal only, *n* = 118 informational only, *n* = 112 informational + persuasive appeal) of approximately equal numbers of men (*n* = 283; 49.7%) and women (*n* = 290, 50.3%) remained. Participants ranged in age from 18 to 44 (*M* = 32.42, *SD* = 6.15), and *n* = 7 not indicating their age. The majority of participants had one or more children (61.0%). The majority of participants resided in the United Kingdom (90.1%), followed by Canada (8.6%), and Australia (1.2%).

### Materials

Three videos were professionally produced in Australia by a media company for use in a social marketing campaign (independent from and prior to the research) to raise awareness of the seriousness of body dissatisfaction; one reflected a narrative approach to communication, one an informational approach, and the other was an explicit persuasive appeal. Experts in the field of body image and eating disorders gave feedback on the scripting and information prior to the production of the videos. Permission to use the materials in the research was granted by the media company.

#### *Narrative approach*

The narrative video (2.48 min) featured a woman in her early thirties engaging in an internal monologue of negative body talk (e.g., “If Mia’s mum needs to lose 5 kilos to get rid of her non-existent cellulite, how many kilos do I need to lose to be part of the short-shorts sports carnival day mothers’ club?!”) while driving in the car with her child. The woman eventually realises the harshness of her self-talk and acknowledges the functionality of her body over its appearance. The woman is white and thin, and was shown sitting. The actor was pregnant at the time of filming but this was not visible within the video as a bag was covering her torso. The narrative video can be viewed at https://vimeo.com/entertainthinkinspire/tmp1.

#### *Informational approach*

The informational video (2.54 min) was comprised of interviews with five body image experts (an academic, a dietician, a medical practitioner, and chief executive officer and education manager of two eating disorders support services) who discussed factual information about body image (e.g., “Body dissatisfaction is a problem across our society; young, old, males, females… body image problems just don’t go away with age, it’s not that you get to some level of enlightenment and say “Ok I’m fine and I’m not worried about these issues anymore”, actually they hang around for a really long time”. The information included topics such as contributing factors, sociocultural appearance pressures, appearance comparison, help-seeking for body dissatisfaction, and the potential usefulness of challenging appearance ideals. All presenters in the video were white and cisgender, and all but one were both a) thin and b) women. One presenter was a larger bodied man. Only the upper bodies, not full-body view of presenters were shown. The video can be viewed at https://vimeo.com/entertainthinkinspire/tmp5.

#### *Persuasive appeal*

The persuasive appeal video (1.20 min) featured the female actor from the narrative approach video presenting a direct appeal to the viewer to increase their awareness and understanding of body dissatisfaction, and question and challenge appearance ideals (e.g., “What can we do about it? I think the first thing is to be aware of it…” “Starting to have conversations within ourselves, within our social circles, within our families, within our community and hopefully then globally”. The actor briefly mentioned her own difficulties with body dissatisfaction. The actor, as previously described, was white and thin, although she was pregnant at the time. This video can be viewed at https://vimeo.com/156214950.

#### *Narrative approach + persuasive appeal*

The narrative approach video was shown followed by the persuasive appeal video (4.08 min), as described above.

#### *Informational approach + persuasive appeal*

The informational approach video was shown with footage edited in from the persuasive appeal video (3.45 min).

### Measures

#### *Demographic questions*

Demographic questions included age, gender (from the following: male, female, other^1^), country of residence, height, weight, and number of children.

#### *Body satisfaction measures*

A series of visual analogue scales (VAS) were used to assess weight satisfaction, shape satisfaction, and muscularity satisfaction before and after viewing the stimulus. Only pre-video exposure data around weight satisfaction, shape satisfaction, and muscularity satisfaction were used in the current analysis solely for the purposes of examining confounding effects, as the focus of the current study is on engagement outcomes. Participants were asked to indicate how satisfied they feel right now, from 0 (*not at all*) to 100 (*very much so*). Previous research has demonstrated that scores from VAS are valid and reliable for assessing body satisfaction [51, 52]. Higher scores indicated higher body satisfaction. Items were used separately in analyses.

Post-video weight satisfaction, shape satisfaction, and muscularity satisfaction are reported elsewhere. Other measures, including mental health literacy, and behavioural intentions, were assessed both pre- and post-exposure but are reported elsewhere [46].

#### *Engagement*

A set of 18 items measured on VAS was developed specifically for the current study to measure general engagement with the videos and topic. Items were informed by previous research examining responses to health campaigns [14, 53–55]. To investigate the factor structure of the engagement assessment items, an exploratory factor analysis was conducted. An initial Principal Components Analysis was conducted to check the suitability of the data for EFA. The results of KMO tests (0.891) suggested the sample size of 576 was sufficient and Bartlett’s test of sphericity, χ^2^(153) = 4056.95, *p* < .001, suggested that there were sufficiently high correlations among items to perform EFA. Four components were identified with an eigenvalue of > 1, which explained 57.96% of the total variance. Parallel analysis using Vivek et al.’s [56] web based engine indicated that three factors should be retained, as indicated by their eigenvalues being greater than the mean eigenvalue. An exploratory factor analysis was conducted to identify item loadings for three components. Oblique rotation demonstrated that the component correlations between the four components were relatively low (ranging from *r* = .01 to *r* = .36), and as such an orthogonal rotation was used. The rotated component matrix is displayed in the supplementary material (Table S1). Internal consistency analyses demonstrated excellent internal consistency for Factor 1 (α = 0.90), acceptable internal consistency for factor 2 (α = 0.62), and factor 3 (α = 0.69). Item deletion was deemed not to result in improvement for Cronbach’s alpha for any of the scales. The three factors explained 52.93% of the variance (see Table S2). These corresponded with affective (compassion), behavioural (interest), and cognitive (relevance) domains of engagement.

##### *Interest*

Eight VAS items identified from the factor analysis were used to measure participant interest (behavioural engagement) in the video and topic. Participants were asked to indicate their interest, from 0 (*not at all*) to 100 (*very much so*). A mean score from items responses was used for the scale score. Higher scores indicated greater interest. Cronbach’s alpha demonstrated excellent internal consistency, with α = 0.90.

##### *Compassion*

Five VAS items identified from the factor analysis were used to measure participant compassion towards people with body dissatisfaction, as well as self-compassion. These VAS items measured affective engagement. Participants were asked to indicate the degree to which the video was respectful, increased other-directed compassion, oversimplified body image issues (reverse scored), increased blame (reverse scored), and was perceived to make other people feel more concerned about their appearance (reverse scored), from 0 (*not at all*) to 100 (*very much so*). A mean score from items responses was used for the scale score. Cronbach’s alpha demonstrated acceptable internal consistency, with α = 0.62. Higher scores indicated greater compassion.

##### *Relevance*

Three items identified from the factor analysis were used to measure relevance of the video and topic, which reflected cognitive engagement. Participants were asked to indicate the degree to which the video was relevant to their own lives, covered an important topic, and they could recognise their own experiences in the video, from 0 (*not at all*) to 100 (*very much so*). A mean score from items responses was used for the scale score. Cronbach’s alpha demonstrated acceptable internal consistency, with α = 0.69. Higher scores indicated greater relevance.

### Procedure

#### Ethics approval

was granted by the La Trobe University Human Ethics Committee, approval number HEC15-116. To reduce the likelihood of a biased sample with high interest in the topic of body dissatisfaction, the true purpose of the study was partially concealed. Participants were invited to take part in a study on health promotion videos and were informed that they would view either a video on body image or self-esteem. The study took place on Qualtrics online survey software, and participants completed the study in an environment of their choosing. Participants provided their consent, then completed pre-exposure measures of body satisfaction. Participants were then randomly assigned to one of the five video conditions previously described. Simple randomisation was performed automatically through the Qualtrics software on an even basis across all five conditions. After watching the video, participants completed measures of interest, compassion, relevance, and demographic variables. Following completion of the study measures, participants were then debriefed on the true aims of the study and given the option to withdraw their data. Participation took, on average, 16.96 min.

### Manipulation check

To ensure that the quality of the videos was reasonably similar across groups, participants were asked five questions, measured on a Likert-type scale from 1 (*Strongly disagree*) to 5 (*Strongly agree*) about whether the video they watched was engaging^2^, humorous, factual, had high production values, and was visually appealing. Items were analysed separately.

### Data Analysis

Analyses were separated by gender as women tend to have higher body dissatisfaction than men, which was verified using an independent samples t-test. Scores on all body satisfaction items were higher in men than in women; all *p* < .01. Missing value analysis revealed no patterns of missing data, with only two variables for each gender group missing 1% or less. As such, participants with missing data on a relevant variable were excluded from that analysis. Pearson correlations were used to inspect whether pre-existing body satisfaction was related to engagement as a confound. For women, weight, muscularity, and shape satisfaction were significantly related to relevance (all *p* < .001); as such, due to the potential for body satisfaction to confound the relationship between videos and engagement (e.g., greater personal relevance for those with low body satisfaction) this was controlled for in the analysis of relevance for women [57]. Thus, to test whether videos produced differences in relevance for women, an ANCOVA was conducted, controlling for pre-existing muscularity, weight, and shape satisfaction. All assumptions of ANCOVA were met. Pre-existing body image scores were not related to compassion or interest for women (all *p* > .05), and as such two univariate ANOVAs were conducted to test whether videos produced differences in compassion and interest. All assumptions of ANOVA were met. For men, pre-existing muscularity, weight, and shape satisfaction were related to relevance. Thus, to test whether videos produced differences in relevance for men, an ANCOVA was conducted, controlling for pre-existing muscularity (*p* < .05), weight, and shape satisfaction (both *p* < .001) as potential confounds. Assumptions of ANCOVA were met. Body image variables were unrelated to interest or compassion for men (all *p* > .05), so two univariate ANOVAs were conducted to test whether videos produced differences in interest. All assumptions of ANOVA were met. Post-hoc power analyses for ANOVA and ANCOVA omnibus tests were conducted using G*Power [58], which revealed that all analyses were sufficiently powered at between 1-β = 0.93 and 0.94 to detect a medium effect of f = 0.25 with α = 0.05. All post-hoc comparisons were examined using Holm corrections to control for Type 1 error.

## Results

### Manipulation checks

To ensure that the quality of the videos was reasonably similar, we examined participants’ responses to the manipulation check questions. A series of one-way ANOVAs examining the differences between the five groups on the manipulation check questions were conducted. Holm corrections were used to account for multiple comparisons. The information and information + persuasive appeal videos were rated more factual than both the narrative and narrative + persuasive appeal videos (*p*s < 0.001). The narrative and narrative + persuasive appeal videos were significantly more humorous than all other conditions (*p*s < 0.001). These findings suggest the manipulation was successful. The production quality was rated significantly higher in the narrative condition, the information condition, and information + persuasive appeal than the persuasive appeal condition (*p* < .001). Additionally, the visual appeal was rated as higher in the narrative only video, the information + persuasive appeal and the narrative + persuasive appeal than the persuasive appeal video (all *p*s < 0.001).

### Descriptive statistics

The means and standard deviations for compassion, relevance, and interest in each video are presented in Table 1. Overall, compassion appeared to be moderate to high among both men and women, across all conditions. Relevance appeared to be moderate to high across all conditions. Interest was moderate to high across all video conditions. Upon inspection of the descriptive statistics, it also appears that scores for men were generally lower than scores for women. Overall, videos appeared to be engaging for the audience.

**Table 1:** *Means and Standard Deviations for Engagement Variables by Condition and Gender*

	Information Only		Information + Persuasive Appeal		Narrative Only		Narrative + Persuasive Appeal		Persuasive Appeal	
	*Men*	*Women*	*Men*	*Women*	*Men*	*Women*	*Men*	*Women*	*Men*	*Women*
Compassion (Range: 0-100)	66.83 *(13.38)*	70.03 *(13.41)*	67.99 *(16.09)*	66.63 *(14.41)*	59.36 *(12.79)*	60.83 *(14.83)*	63.25 *(12.84)*	67.12 *(15.31)*	65.19 *(12.04)*	71.05 (13.23)
Relevance (Range: 13.33–100)	69.35 *(18.22)*	79.63 *(15.72)*	66.57 *(17.43)*	78.22 *(14.08)*	60.28 *(15.51)*	73.65 *(16.69)*	63.19 *(17.96)*	79.88 *(17.49)*	70.09 *(15.49)*	76.04 *(18.43)*
Interest (Range: 33–100)	59.28 *(19.14)*	67.83 *(16.87)*	60.60 *(21.60)*	68.61 *(13.85)*	56.35 *(18.29)*	66.94 *(18.73)*	60.73 *(18.33)*	71.84 *(16.33)*	57.78 *(18.65)*	64.49 *(20.12)*

### Engagement in videos among women

All descriptive statistics for ANOVAs and ANCOVA analyses for women examining the differences in engagement by video condition are displayed in Table 2.

**Table 2:** *Means and Standard Errors from ANOVAs and ANCOVA for Differences between Video Condition Engagement in Women*

*Variable*	*Information Only* *n = 62*	*Information + Persuasive Appeal* *n = 58*	*Narrative Only* *n = 58*	*Narrative + Persuasive Appeal* *n = 50*	*Persuasive Appeal* *n = 61*	*Pairwise comparisons*
Compassion	70.03 (1.81)	66.63 (1.87)	60.82 (1.87)	67.12 (2.01)	71.05 (1.82)	PA, IN > NA
Relevance^a. b^	4.28 (0.21)	4.46 (0.22)	4.87 (0.21)	4.15 (0.23)	4.58 (0.21)	ns
Interest^b^	5.55 (0.20)	5.56 (0.21)	5.60 (0.21)	5.12 (0.23)	5.77 (0.21)	ns

Note: ^a^ Shape satisfaction, muscularity satisfaction, and weight satisfaction were the covariates, and means are estimated marginal means. ^b^Transformed using a square root and reflect transformation. IN = Information only, IN + PA – information + persuasive appeal, NA – narrative-only, NA + PA – narrative + persuasive appeal, ns – non-significant, PA – Persuasive appeal

#### *Interest*

As measures of pre-test muscularity, weight, and shape dissatisfaction were unrelated to interest in women, a univariate ANOVA was conducted to examine the differences between video conditions on interest. There was no significant effect of video condition on interest, *F*(4, 285) = 1.22, *p* = .30, partial η^2^ = 0.02.

#### *Relevance*

As measures of pre-test weight satisfaction, shape satisfaction, and muscularity satisfaction were related to relevance in women, an ANCOVA controlling for these variables was conducted to examine the differences between video conditions on relevance. Pre-test weight satisfaction (*F* (1, 279) = 42.38, *p* < .001, partial η^2^ = 0.13), and shape satisfaction (*F* (1, 279) = 0.9.34, *p* = .002, partial η^2^ = 0.03) were significantly related to relevance; but muscularity satisfaction (*F* (1, 279) = 2.39, *p* = .12, partial η^2^ = 0.01) was not. There was no significant effect of video condition on relevance, *F* (4, 287) = 1.61, *p* = .17, partial η^2^ = 0.02.

#### *Compassion*

As measures of pre-test muscularity, weight, and shape dissatisfaction were unrelated to compassion in women, univariate ANOVA was conducted to examine the differences between video conditions on compassion. There was a significant effect of video condition on compassion, *F* (4, 284) = 4.67, *p* = .001, η^2^ = 0.06. Holm corrections revealed that the persuasive appeal video (M = 71.05, SE = 1.82) resulted in significantly greater compassion than the narrative only video (M = 60.82, SE = 1.87), adjusted *p* = .002. The information only video (M = 70.03, SE = 1.81) resulted in significantly greater compassion than the narrative only video (M = 60.82, SE = 1.87), adjusted *p* = .005. No other significant differences were found.

### Engagement in videos among men

All descriptive statistics for ANOVAs and ANCOVA analyses for men examining the differences in engagement by video condition are displayed in Table 3.

**Table 3:** *Means and Standard Errors from ANCOVAs and ANOVA Examining Differences by Video Condition on Engagement in Men*

*Variable*	*Information Only* *n = 56*	*Information + Persuasive Appeal* *n = 54*	*Narrative Only* *n = 56*	*Narrative + Persuasive Appeal* *n = 58*	*Persuasive Appeal* *n = 57*	*Pairwise comparisons*
Compassion	66.82 (1.80)	67.99 (1.83)	59.36 (1.78)	63.25 (1.75)	65.19 (1.78)	IN + PA, IN, PA > NA
Relevance^a^	70.61 (2.18)	66.93 (2.21)	59.93 (2.18)	62.50 (2.14)	69.57 (2.16)	IN > NA, NA + PA; PA > NA, NA + PA. IN + PA > NA.
Interest^b^	6.10 (1.58)	5.94 (1.75)	6.36 (1.51)	6.00 (1.54)	6.24 (1.50)	ns

Note: ^a^ Shape satisfaction, weight satisfaction, and muscularity satisfaction were the covariates, and means are estimated marginal means reported with standard errors. ^b^Variable was transformed using the square root procedure IN = Information only, IN + PA – information + persuasive appeal, NA – narrative-only, NA + PA – narrative + persuasive appeal, ns – non-significant, PA – Persuasive appeal

#### *Interest*

As measures of pre-test muscularity, weight, and shape dissatisfaction were unrelated to interest in men, a univariate ANOVA was conducted to examine the differences between video conditions on interest. There was no significant effect of video condition on interest, *F* (4, 277) = 0.66, *p* = .62, η^2^ = 0.01.

#### *Relevance*

As measures of pre-test weight satisfaction, shape satisfaction, and muscularity satisfaction were related to relevance in men, an ANCOVA controlling for these variables was conducted to examine the differences between video conditions on relevance. Pre-test shape satisfaction was significantly related to relevance; *F* (1, 273) = 7.44, *p* = .007, partial η^2^ = 0.03, but weight satisfaction (*F* (1, 273) = 0.34, *p* = .56, partial η^2^ < 0.01) and muscularity satisfaction (*F* (1, 273) = 1.96, *p* = .16, partial η^2^ = 0.01) were not. There was a significant effect of video condition on relevance, *F* (4, 273) = 4.45, *p* = .002, partial η^2^ = 0.06.

Holm corrections revealed that the information only video (M = 70.61, SE = 2.18) resulted in significantly greater relevance than the narrative only video (M = 59.93, SE = 2.18; adjusted *p* = .005) and the narrative + persuasive appeal video (M = 62.50, SD = 2.14; adjusted *p* = .027). The persuasive appeal (M = 69.57, SE = 2.16) resulted in significantly greater relevance than the narrative only (M = 59.93, SE = 2.18; adjusted *p* = .008), and the narrative + persuasive appeal video (M = 62.50, SE = 2.14; adjusted *p* = .042). The information + persuasive appeal video (M = 66.93, SE = 2.21) resulted in significantly greater relevance than the narrative only video (M = 59.93, SE = 2.18; adjusted *p* = .025). No other significant differences were found.

#### *Compassion*

As measures of pre-test muscularity, weight, and shape dissatisfaction were unrelated to compassion in men, a univariate ANOVA was conducted to examine the differences between video conditions on compassion. There was a significant effect of video condition on compassion, *F* (4, 278) = 3.57, *p* = .007, partial η^2^ = 0.05. Holm corrections revealed that the information and persuasive appeal (M = 67.99, SE = 1.83, adjusted *p* = .003), information only (M = 66.82, SE = 1.80, adjusted *p =* .006), and persuasive appeals (M = 65.19, SE = 1.78, adjusted *p =* .022) all resulted in significantly greater compassion than the narrative only video (M = 59.36, SE = 1.78). No other significant differences were found.

## Discussion

The aim of this study was to compare participants’ reported engagement with different forms of persuasive communication about body dissatisfaction, focusing on informational, narrative, and direct persuasive appeal. We proposed two research questions. The first was whether a particular persuasive approach resulted in superior engagement over other approaches in the context of body dissatisfaction mental health literacy. It was found that there was some advantage for both persuasive appeals and information-only approaches over a narrative approach for both gender groups, and some advantage for the information approach with the addition of a persuasive appeal for men. The second was to understand whether different persuasive communication approaches impacted cognitive, affective, and behavioural domains of engagement differently. It was found that there were some domains impacted differently depending on the communication approach, in particular for cognitive and affective domains.

Overall, while all communication types were engaging, there appeared to be some advantage for persuasive appeals and informational videos across both men and women. This is only partially consistent with the previous literature around persuasive communication, which has found benefits overall for both informational and narrative approaches [35, 36]. Although ratings of compassion and relevance were reasonably high in all conditions, the current study’s findings clarify that narrative approaches were slightly less likely to promote engagement relative to other approaches within the context of body image social marketing. One explanation for this finding is that, while it is quite common to have knowledge of a person who struggles with body dissatisfaction (as in the narrative video), it may be less common for people to receive evidence-based and statistical information about body dissatisfaction or hear a persuasive appeal around body dissatisfaction, as indicated by poor levels of mental health literacy [7]. As such, the novelty of these video approaches may have resulted in higher ratings for engagement. An alternative explanation is that the use of humour in the narrative context was ineffective for promoting engagement, or potentially clouded the message, relative to the other videos. Non-humorous approaches should be investigated in future studies to examine whether this effect can be replicated. It should be noted that all approaches were equally effective for body satisfaction outcomes in our previous work [46]; and narrative approaches were not associated with poorer outcomes for interest and relevance for women, or interest for men, relative to other videos in the current study.

The benefits observed from viewing a persuasive appeal (relative to the narrative videos) are a novel finding, as to our knowledge the effects of viewing a standalone persuasive appeal were previously unexamined in the literature. This finding may indicate that the nature of a persuasive appeal is appropriate for use on social media or in other forms of media. The message is short, clear, and action-based, giving viewers a specific call to action to learn more about body dissatisfaction as an important mental health issue. This appeal bears resemblance to very successful strategies commonly used in social media (e.g. call to action) which are intended to generate engagement [59]. It is interesting that the addition of a persuasive appeal did not appear to boost the engagement response to the narrative-only video, despite apparent benefits for adding a persuasive appeal to a narrative in previous literature [18]. One explanation for this is continuity between the first and second video message (e.g., the transition between the tone and other characteristics of the narrative video and the persuasive appeal video) – perhaps the videos seemed too disjointed and participants could not see consistency in the messages. This may be a characteristic to examine in future studies. The persuasive appeal did not appear to boost engagement in the informational video, although for men, it was associated with equally high compassion and relevance as the information only video. In line with the suggestion for examining the impact of persuasive appeals on narrative videos, research may further knowledge in this area by examining the effect of a persuasive appeal with greater continuity.

Different impacts on domains of engagement were found, which were partially consistent with literature on processing communication [43, 44]. Interestingly, for men, the findings for informational videos affecting cognitive engagement were consistent with Willoughby and Liu [38], who found that informational approaches to communication may result in greater elaboration. This may reflect central or systematic processing [38, 60]. While this advantage for the cognitive domain of engagement was not observed for women, elaboration of the content may have impacted affective responses. Our previous analyses [46] for these videos indicated that informational approaches were more successful in producing perceptions of body dissatisfaction as serious relative to narrative approaches. This finding may be aligned with this study’s results of greater compassion in the informational and persuasive appeal condition compared with the narrative condition, as empathy for someone with body dissatisfaction is related to recommending help-seeking – i.e., that the condition is serious [7]. As the compassion factor also measured self-compassion, informational videos may help people to recognise the seriousness of their own body dissatisfaction.

Inconsistent with theories of processing and attitude bases [33, 41, 44, 60], narrative approaches did not appear to impact compassion to a greater extent than other approaches for either men or women. This may be explained by the humorous content of the narrative video, and as such future research should examine effects of narrative persuasion that do not use humour. It is notable, however, that persuasive appeals and informational videos (whether or not they contained a persuasive appeal) improved compassion in men relative to narrative videos. Considering the high levels of stigmatization towards eating disorders and body image difficulties observed among men in previous literature [61], persuasive appeals and informational videos are strategies that researchers may like to assess for their effectiveness in increasing compassion. Considering that the narrative video depicted a woman’s experience, this may have been less effective in men than other video types, as men may have been less able to relate to the experience depicted in the video. Future research may wish to examine the effects of narrative videos featuring men’s stories.

Notably, although short videos showed differences for compassion and relevance (for men) and compassion (for women), no videos appeared to produce superior results in all domains of engagement across genders. This finding is not especially concerning in itself, particularly when examining the findings for relevance and interest in women (i.e., both relevance and interest were fairly high among women regardless of video condition). It should be of note, however, that interest (i.e., behavioural engagement) was moderate among men, which may indicate less intent to engage with body image content more generally as body dissatisfaction and eating disorders are perceived to be feminine issues [61]. Reasons for this belief previously identified in research include lack of representation of men’s body dissatisfaction in media narratives [9], and masculine norms preventing men from discussing their dissatisfaction [9, 62, 63]. Future research could examine whether videos designed specifically with men in mind, using the same approaches used in the current study, may impact engagement differently.

The findings of this study have implications for social marketing campaigns within the body image field, and research into their efficacy. First, it is clear that further research exploring the impact of persuasive appeals should be conducted, as they were more engaging than narrative approaches within some contexts. Second, contextualising the current study’s findings with our previous work, social marketing campaigns that aim to educate and promote awareness through engagement may take a different approach from those aimed at improving body satisfaction. There may be some advantages to informational and persuasive appeals relative to narrative approaches within the context of engagement. Campaigns may wish to use expert information, or direct appeals to the audience, particularly to generate interest online. Third, research should further examine the impact of particular persuasive communication approaches on domains of engagement. For instance, a call to action may best be used to impact affective engagement. Further examination of persuasive communication approaches on specific domains of engagement would be beneficial for social marketers and researchers.

### Strengths and Limitations

A strength of this study was its generalisability; a mixed-age, community sample of men and women was used, which is not common in evaluating social marketing or anti-stigma campaigns for body image and eating disorders [64]. The measurement of multiple domains of engagement is also a strength; previous research on social marketing engagement has tended to focus on sharing and ‘liking’ content. It should be noted, however, that our behavioural measures were *intended* behaviour rather than actual behaviour, and it would be beneficial to evaluate such messages including a measure of actual behaviour – for example, whether participants click on a link to visit a website about body image.

Further limitations were that engagement was examined at one point in time, which precluded examination of change in engagement resulting from video viewing, and that the use for the cover story likely did not fully conceal the purpose of the study. In addition, the standalone persuasive appeal video was rated as less visually appealing and with lower production quality than the other videos, which may have impacted interest and attention to the videos. Another limitation is the varying length of the videos, in particular the difference in viewing time between the persuasive appeal only video, and the conditions to which the persuasive appeal video was added. As such, the effects of video length from video content may be difficult to separate. Although all videos were short in duration, future research should consider using equivalent length videos in all conditions. An additional limitation is around cultural relevance; although we collected country of residence, we did not collect information on other demographic characteristics; which may be of relevance considering academics and actors appearing in the videos were white. Future research examining engagement in such videos may wish to collect information such as cultural background, to better assess generalisability of the research, and aim to ensure the cast is not all or majority white to better reflect a community sample’s characteristics. Finally, a limitation is that most people in the video were thin; this may have unintentionally reinforced the idea that body dissatisfaction is only a problem when the person’s body is smaller; further research should examine the impact and potential benefits of educating the public that body dissatisfaction has a negative impact at all sizes, the issue of weight stigma, and attempt to include more size diversity.

## **Conclusion**

The current study demonstrated that attempts to raise awareness of body dissatisfaction through persuasive communication were highly engaging in relation to behavioural (interest), affective (compassion) and cognitive (relevance) domains. Although all videos were rated as highly engaging, persuasive appeals and informational approaches were rated more highly on some engagement domains by men and by women. These findings suggest that video messages that are demonstrated to be effective in increasing perceptions of the seriousness of body dissatisfaction, as our previous research demonstrated [46], may also lead to greater dissemination of information and education about the topic.

## Electronic supplementary material

Below is the link to the electronic supplementary material.

Supplementary Table S1 and S2

## Data Availability

The datasets generated and/or analysed during the current study are not publicly available as this was not a condition outlined to our participants, but are available from the corresponding author on reasonable request.

## Footnotes

1 Note – The authors recognise that this is not currently an ideal way of asking about gender identity, and use and advocate for more inclusive and affirming ways of asking about gender identity.

2 This was a single-item measure of engagement for manipulation check purposes, and thus was not intended to measure the specific engagement we have examined in this study.
