# Supplementary material for: Engagement with body image health promotion videos in adult men and women: differences between narrative, informational, and persuasive appeal approaches
Source: BMC Psychol. 2023 Mar 29;11:86. doi: 10.1186/s40359-023-01120-7 (PMC10061748; doi:10.1186/s40359-023-01120-7)
Supplement: Supplementary file 1 — Supplementary Table S1 and S2 [file 40359_2023_1120_MOESM1_ESM.docx]

To investigate the factor structure of the engagement assessment items, an exploratory factor analysis was conducted. An initial Principal Components Analysis was conducted to check the suitability of the data for EFA. The results of KMO tests (.891) suggested the sample size of 576 was sufficient and Bartlett’s test of sphericity, χ^2^(153) = 4056.95, *p*<.001, suggested that there were sufficiently high correlations among items to perform EFA. Four components were identified with an eigenvalue of >1, which explained 57.96% of the total variance. Parallel analysis using Patil et al.’s (2017) web based engine indicated that three factors should be retained, as indicated by their eigenvalues being greater than the mean eigenvalue. An exploratory factor analysis was conducted to identify item loadings for three components. Oblique rotation demonstrated that the component correlations between the four components were relatively low (ranging from *r*=.01 to *r*=.36), and as such an orthogonal rotation was used. The rotated component matrix is displayed in supplementary table 1. Internal consistency analyses demonstrated excellent internal consistency for Factor 1 (α=.90), acceptable internal consistency for factor 2 (α=.62), and factor 3 (α=.69). Item deletion was deemed not to result in improvement for Cronbach’s alpha for any of the scales. The three factors explained 52.93% of the variance (see Supplementary Table 2). These corresponded with affective (compassion), behavioural (interest), and cognitive (relevance) domains of engagement.

Table S1. Findings from Initial Principal Components Analysis on Engagement Items.

|  | *Factor 1* | *Factor 2* | *Factor 3* | *Factor 4* |
| --- | --- | --- | --- | --- |
| It is important to focus on the issue | **.46** | .04 | **.53** | .17 |
| The topic is relevant to my life | **.48** | -.30 | **.65** | .12 |
| Recognise the issue from my personal experiences | **.58** | -.18 | **.47** | -.05 |
| Recognise the issue from my observations of others | **.55** | .12 | .15 | -.10 |
| Likely to reflect | **.72** | -.13 | -.15 | .06 |
| Level of self-reported engagement in video | **.73** | -.23 | -.27 | .02 |
| Want to learn more | **.77** | -.23 | -.05 | .13 |
| Increase blame towards people with body dissatisfaction (reverse scored) | .29 | **.65** | .19 | .10 |
| Makes body image issues seem simpler than they really are (reverse scored) | .01 | **.48** | .08 | **.57** |
| Increase compassion towards people with body dissatisfaction | **.62** | .37 | -.01 | -.19 |
| Portray people with body dissatisfaction in a respectful manner | **.51** | **.49** | -.04 | -.27 |
| Make people more concerned about their appearance (reverse-scored) | .10 | **.57** | .07 | .05 |
| Make people more movtivated to be kind to themselves in relation to their appearance | **.64** | .24 | -.16 | -.28 |
| Have a positive impact on viewers | **.74** | .38 | -.15 | -.19 |
| Share video with one or more people | **.72** | -.29 | -.26 | .19 |
| Would you believe that this video has been viewed over 5 million times on YouTube? | .09 | -.29 | .27 | **-.62** |
| Read an article on the topic after watching video | **.76** | -.18 | -.10 | .16 |
| Discuss the topic/content of this video in the next month | **.75** | -.27 | -.20 | .16 |
| Loadings | Eigenvalue 6.11, 33.93% variance | Eigenvalue 2.09, 11.63% variance | Eigenvalue 1.33, 7.37% variance | Eigenvalue 1.10, 6.11% variance |

Note: Items with loadings above .40 are presented in bold.

Table S2. Findings from Exploratory Factor Analysis on Engagement Items.

|  | *Factor 1*  *Interest* | *Factor 2*  *Compassion* | *Factor 3*  *Relevance* |
| --- | --- | --- | --- |
| Level of self-reported engagement in video | **.80** | -.03 | .09 |
| Share video with one or more people | **.80** | -.08 | .12 |
| Discuss the topic/content of this video in the next month | **.80** | -.04 | .18 |
| Read an article on the topic after watching video | **.75** | .05 | .25 |
| Want to learn more | **.74** | .02 | .31 |
| Likely to reflect | **.73** | .08 | .18 |
| Have a positive impact on viewers^a^ | **.62** | **.56** | .05 |
| Make people more motivated to be kind to themselves in relation to their appearance^a^ | **.59** | **.41** | .05 |
| Recognise the issue from my observations of others | .39 | .30 | .31 |
| Increase blame towards people with body dissatisfaction (reverse scored) | .02 | **.73** | .10 |
| Portray people with body dissatisfaction in a respectful manner | .35 | **.62** | .03 |
| Make people more concerned about their appearance (reverse-scored) | -.07 | **.58** | -.05 |
| Increase compassion towards people with body dissatisfaction^a^ | **.46** | **.54** | .13 |
| Makes body image issues seem simpler than they really are (reverse scored) | -.14 | **.47** | -.05 |
| The topic is relevant to my life | .21 | -.04 | **.84** |
| Recognise the issue from my personal experiences | .34 | .08 | **.69** |
| It is important to focus on the issue | .16 | .26 | **.64** |
| Would you believe that this video has been viewed over 5 million times on YouTube? | .01 | -.21 | .34 |

Note: Items with loadings above .40 are indicated in bold. ^a^Higher loading was used for this item.
